# Traumatic enucleation of the left globe after a road traffic accident – A case report of an uncommon occurrence in maxillofacial trauma

**DOI:** 10.1016/j.ijscr.2020.12.011

**Published:** 2020-12-05

**Authors:** Antonia Taiane Lopes de Moraes, Martha Caroline Auzier Quaresma, Thais Freitas Silva, Naama Waléria Alves Sousa, Silvio Augusto Fernandes Menezes, Andre Luis Ribeiro Ribeiro, João de Jesus Viana Pinheiro

**Affiliations:** a Department of Oral Pathology, School of Dentistry, Federal University of Para, Avenue Augusto Correa, 01. Belem, Para, Brazil; b School of Dentistry, University Center of Pará (CESUPA), Belém, Pará, Brazil; c Private Practice, INCOM – Instituto de Cirurgia Oral e Maxilofacial (www.institutoincom.com), Belém, Pará, Brazil; d Department of Periodontics, School of Dentistry, University Center of Pará (CESUPA), Belém, Pará, Brazil

**Keywords:** OZC, orbit-zygomatic complex, DM II, type II diabetes mellitus, OGI, open globe injury, Traumatic enucleation, Facial trauma, Orbital fractures, Case report

## Abstract

**Introduction:**

Serious injuries of the globe are uncommon in closed maxillofacial trauma, as the anatomical configuration of the orbit offers important protective mechanisms against external trauma. Thus, the objective of this work is to report a case of a rare traumatic enucleation of the globe resulting from maxillofacial blunt trauma.

**Presentation of case:**

A 42-year-old man was hit by a car with a major complaint of facial pain. The patient had several facial fractures including: Le Fort I, naso-orbit-ethmoidal, anterior sinus wall and an exposed fracture of the orbit-zygomatic complex (OZC). The patient underwent to open reduction and internal fixation of facial fractures and enucleation of the left globe, however, evolved with postoperative infection and complications associated with, a so far unknown, type 2 diabetes. After secondary surgeries for removal infected bones and diabetes control, all injuries were fully healed, and the patient remained with major sequels.

**Discussion:**

In this case, we hypothesized a high-energy trauma resulting in multiple facial fractures, especially involving the OZC, dislocated the thick lateral wall of the orbit within the orbital cavity and reduced the orbital volume. This resulted in an exaggerated increase in intraorbital pressure, which exceeded the capacity of all anatomical protective mechanisms of the globe, and pushed the globe outwards, causing a complete avulsion.

**Conclusion:**

Besides all anatomical structures to protect the globe, rarely high energy maxillofacial trauma can cause severe damage to the globe resulting in loss of vision and globe avulsion.

## Highlights

• Sometimes the orbital mechanisms to protect the globe fail.
• Although rare, globe avulsion can occur after maxillofacial blunt trauma.
• Despite all efforts, permanent major sequels can occur after facial trauma.

## 1 Introduction

The globe is located in the orbital cavity, surrounded by thin bone walls, especially, the medial and inferior walls. Intraconal and extraconal orbital fat pads offer extra protection from external trauma allowing the displacement of the globe and absorbing part of the energy of the trauma [[1], [2], [3]]. When an orbital fracture occurs, the thin orbital walls can fracture, displacing part of the orbital content to the paranasal sinuses, reducing the energy transferred directly to the globe. Together, these natural anatomical mechanisms act as a real protection from major injuries to the globe, reason why severe globe damage is uncommon in maxillofacial blunt trauma, despite the high prevalence of orbitozygomatic complex (OZC) [3]. There is a wide spectrum of eye injuries that are observed more frequently in facial trauma [4], but the traumatic avulsion of the globe is an uncommon complication of blunt facial trauma and few cases have been reported in the literature [5].

Although OZC fractures are commonly associated with high-energy blunt trauma, such as in road traffic accidents [3], the protective anatomical mechanisms of the eye appear to be quite effective in preventing more severe injuries, making the avulsion of the globe a very rare event [6,7]. The avulsion of the globe occurs as a result of total disruption of the extraocular muscles and optic nerve, by moving the eye out of orbit [5]. Self-avulsions have also been reported, mainly in patients with psychotic disorders or drug addicts [5,8]. These situations are quite severe and always result in complete loss of vision of the affected eye.

In this paper, we report a case of a traumatic enucleation of the left globe on a pedestrian involved in a road traffic accident who presented multiple facial injuries. Exposed panfacial fractures that evolved to late complications were seen, calling the attention to the importance of preventing traffic accidents and how maxillofacial trauma can result in severe sequels. The patient was treated in a level III public trauma center and diagnosis and therapeutic procedures are discussed.

## 2 Presentation of case

**Fig. 1:**
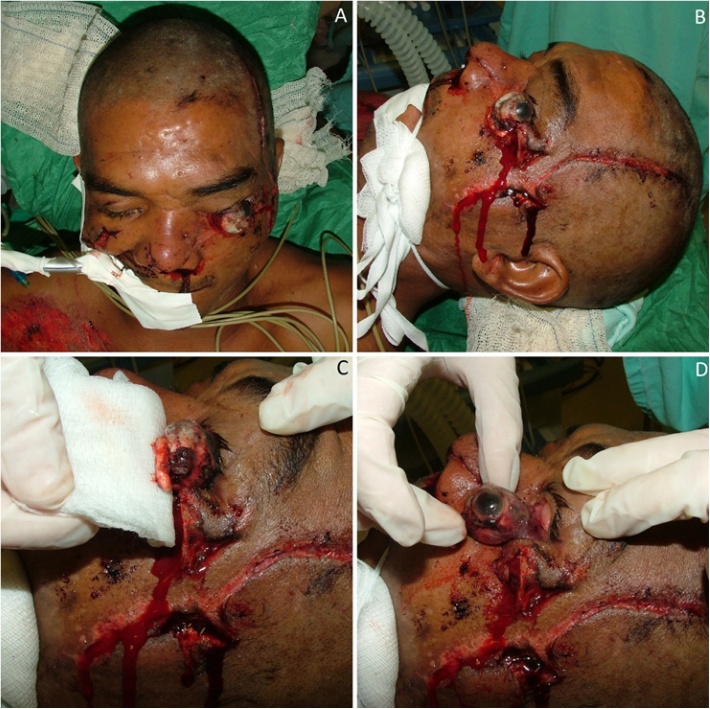
Pre-operative clinical aspect of the patient. Frontal (A) and lateral (B) view showing a dislocated left globe. Lacerated optic nerve (C) and extraorbital muscles (D).

A 42-years-old mixed male was involved in a hit-and-run car accident presenting severe multiple injuries. The patient was taken to a level III public trauma center by ambulance. The patient was seriously injured with exposed facial fractures appearing as his main concerns. Injuries included a panfacial fracture (Le Fort I in maxilla, nasal, anterior frontal sinus wall and an exposed orbitozygomatic complex (OZC) fracture), traumatic enucleation of the left eye, multiple skin injuries, and exposed left leg fracture. The lateral and medial rectus muscles as well as the optic nerve were-sectioned. A large lacerated facial wound was observed in the left temporal region extending to the cheek and other multiple smaller wounds around the orbital region. The globe was found outside the orbit with a laceration due to an open injury that resulted in loss of normal tonicity (Fig. 1A–D). An informed consent form was signed by the patient to report the case and showing his pictures. The work has been reported in line with the SCARE 2018 criteria [9]. This case report is part of an umbrella research protocol approved by the ethics committee of the University Center of Pará (approval number 524680).

**Fig. 2:**
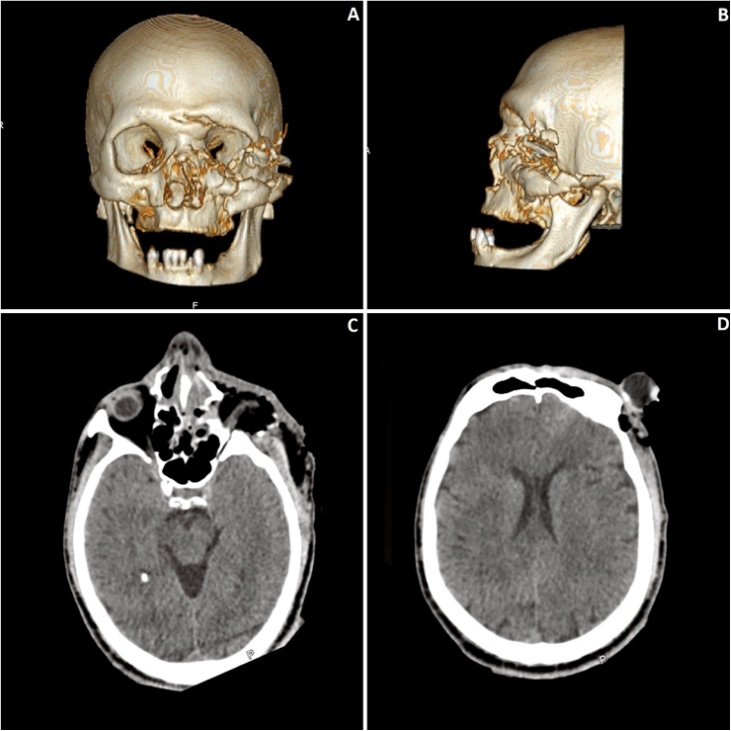
Pre-operative CT scan. Frontal (A) and lateral view (B) of a 3D CT reconstruction showing a panfacial fracture with comminution of the left orbitozygomatic complex. Axial cuts showing a sectioned optic nerve (C) and a the displaced left globe (D).

On computed tomography scan, a comminuted left OZC fracture was observed, the globe was displaced anteriorly, and the optic nerve was teared. A Le Fort I, anterior frontal sinus wall and the naso-orbito-ethmoid fracture were also observed (Fig. 2A–D). An exposed left leg fracture was also seen (not shown).

The patient was taken to the operating room for emergency surgery and taken to multidisciplinary treatment. Closed reduction and external fixation of the left leg fracture was performed by the assistant orthopedist with more than 5 years of experience in accident and emergency. A senior ophthalmologist completed the enucleation of the left globe after considering it as non-salvageable.

**Fig. 3:**
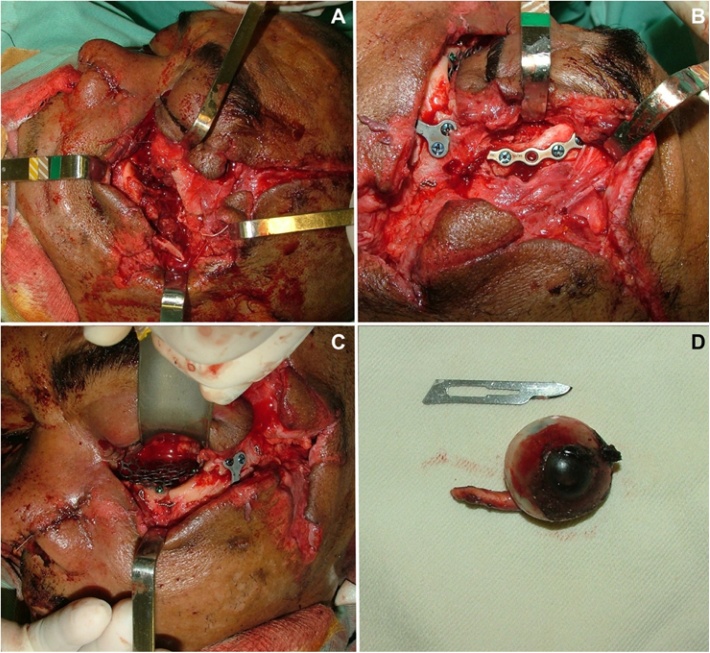
Intraoperative surgical view. Lateral view showing the comminutive orbitozygomatic fracture (A). Open reduction and internal fixation of the orbitozygomatic fracture (B) and reconstruction of orbital floor with titanium mesh (C). Removed left globe (D).

**Fig. 4:**
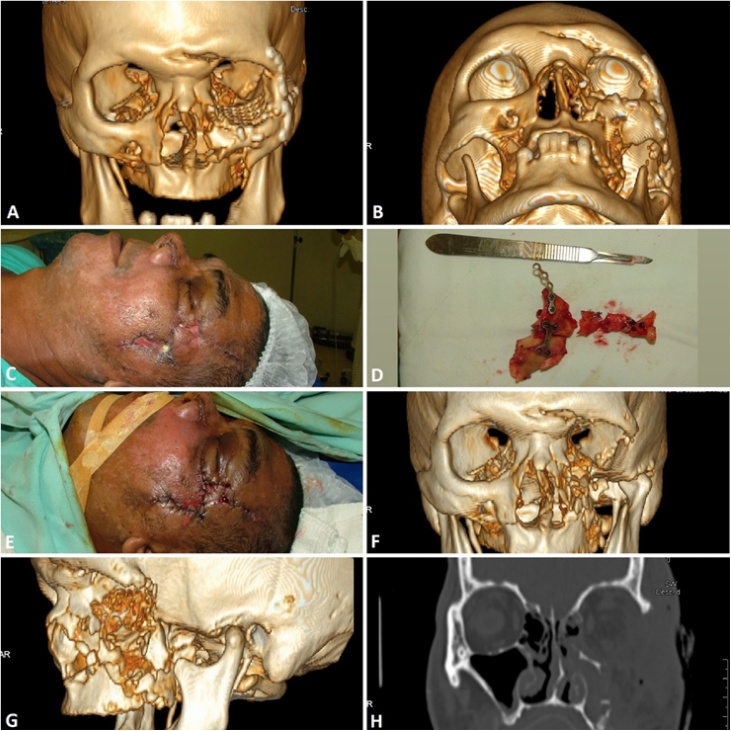
Immediate postoperative 3d reconstructive CT scan. Showing orbitozygomatic (A) and zygomatic arch (B) reconstruction. Clinical view of post-operative infection (C), removed plate and screws with devitalized bone fragments (D), and final appearance after second surgery (E). Post-operative CT scan (F-G). Frontal (F) and lateral (G) 3D reconstruction and coronal (H) cut of CT scan view showing the massive orbital defect.

Maxillofacial treatment included a surgical debridement of the wounds, removal of small bone fragments and open reduction and internal fixation with plates and screws using the wounds as surgical approach (Fig. 3A–F). Reconstruction of the OZC complex was carried out, nasal fracture was treated with closed reduction and anterior nasal packing. All maxillofacial fractures were treated by an experienced and head of the maxillofacial service. There was no displacement of frontal and maxillary fracture (patient had no maxillary teeth), and these fractures were both treated conservatively (Fig. 4A and B).

The patient was treated with intravenous antibiotics (cefazolin 2 g prophylactic and maintained with cephalothin 1 g every 6 h). Blood tests showed anemia due to blood loss that did not require blood transfusion (hemoglobin was 10.2 g/dL and hematocrit 32.4%), however, glucose test was high, 204 g/dL.

No previous medical data was available, and the patient was unaware of any previous medical chronic condition. In-hospital investigation revealed that the patient has type II diabetes mellitus (DM II), which was unknown and uncontrolled before the trauma. The patient was collaborative with all measures to control his condition. Besides that, by the fifth postoperative day, the patient began to show signs of infection on the face and the on left leg. Despite all measures to control the infections (antibiotics, local cleaning, diabetes control), the infection continued to evolve and required secondary surgeries. Maxillofacial management required removal of all plates and screws, titanium mesh and devitalized bone fragments (Fig. 4C–E). Almost the entire zygomatic bone along with the inferior orbital rim and zygomatic arch were removed (Fig. 4F–H). Amputation of the left leg was carried out 4 weeks after admission.

**Fig. 5:**
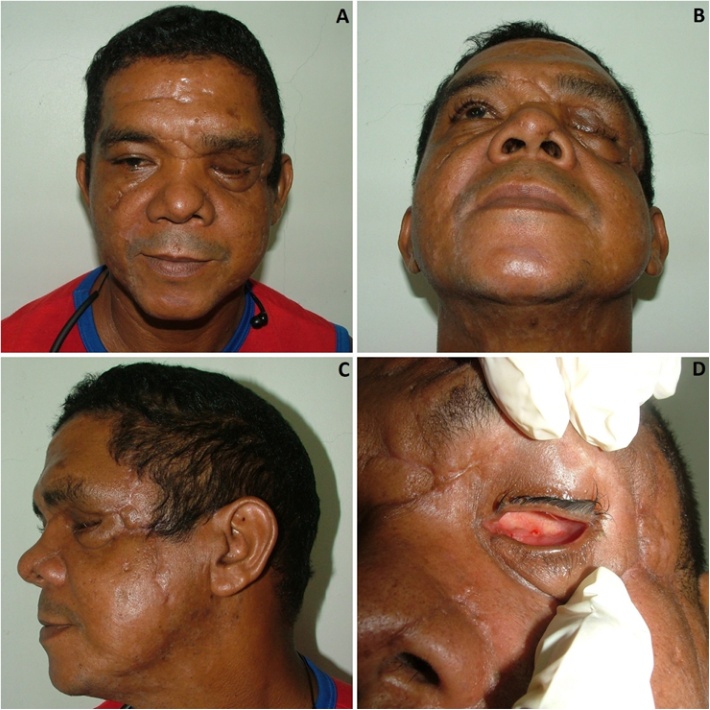
Six month post-operative aspect of the patient. Frontal, submentual and lateral view showing a facial assyetry, orbitozygomatic defect, and eyelid lymphedema. Intraorbital view after healing of the left eye enucleation (D).

After secondary surgeries, infection was controlled, and the patient was discharged 45 days after the admission. The patient was followed-up monthly for a total of six months post-operative in an outpatient clinic. After this period, complete healing of wounds, absence of local infection, and a noticeable facial asymmetry was observed. There was a depression on the left zygomatic region, maintenance of an eyelid edema due to a lymphedema (Fig. 5A–D). The patient was referred to an ophthalmic plastic and reconstructive center for evaluation, but doctors did not find the required support for adaptation of the prosthetic eye and the patient reject any further surgery, choosing to stay with the current sequels. In general, after the severe trauma, sequels and discovery of his untreated type II diabetes, the patient was grateful to be alive and any reconstructive surgery was rejected by the patient.

## 3 Discussion

In this paper was reported an uncommon case of traumatic enucleation of the left globe that evolved with further sequels associated with a maxillofacial blunt trauma. Although several mechanisms have been described as protective to the globe from severe injuries [[1], [2], [3]], in a few situations they fail, and major injuries can occur [2].

Some theories have been proposed to elucidate the mechanisms of globe avulsion, but they are mainly explained case-by-case and can’t be extrapolated to all situations [10]. In this case, we hypothesized that a high-energy trauma resulting in multiple facial fractures, especially those involving the OZC, displaced the thick lateral orbit wall inside the orbital cavity and reduced the orbital volume. This resulted in a massive increase of the intraorbital pressure [10], which extrapolated the capability of all protective anatomical mechanisms of the globe, and pushed the globe outwards, causing a complete avulsion with rupture of the extraocular muscles and optic nerve.

Avulsion of the globe can be classified as incomplete when only the optic nerve is teared damaged, and complete when there is disruption of the extraocular muscles and optic nerve, resulting in a total avulsion of the globe [5].

The surgical approach in severe globe injuries can be very controversial. Some authors suggest that the restoration and maintenance of the globe can offer psychological and aesthetic benefits, in addition to improving the subsequent result of an ocular prosthesis [6,7]. However, in cases when the globe is severely damaged, such as in this case, functional and anatomical rehabilitation of the globe is almost impossible, and enucleation is a viable option [5].

Although rare, sympathetic ophthalmia, which is a serious condition that can lead to blindness of the non-affected eye [11], is reported as a complication of open globe injury (OGI), and primary enucleation is a treatment option. It is believed to be secondary to the development of an autoimmune reaction to ocular antigens that are exposed during the traumatic or surgical event. It triggers an immune response mediated by T cells that attack the contralateral eye [7,11,12]. In a large retrospective study, the prevalence of enucleation in OGI was 6.2% [13]. In this report, the cause of enucleation was the blunt trauma and the ophthalmologist completed the enucleation.

Definitive treatment of open fractures of limbs is often delayed preventing post-operative infection. On the other hand, the face has a rich blood supply and immediate open reduction and internal fixation of open fractures is recommended [14]. Although in this report, the patient evolved with post-operative infection, it may be resulted of a complex set of events that include an uncontrolled DM II, severe damage of the soft tissues and bone fragments, and high load of microbial contamination.

In this paper we have reported an unexpected case of traumatic enucleation of the globe after a severe maxillofacial trauma. Although the OZC is one of the most fractured regions in maxillofacial fractures, anatomical mechanisms exist to protect the globe and optic nerve from serious damage, reason why this case is considered uncommon and exemplify the potential complications of this type of injury.

The patient developed many complications including loss of the left globe, post-operative infection, loss of entire zygomatic bone and part of the orbit, even though the case was managed by standard therapies. Prevention is the most important measure to avoid this type of sequels, which are rare in maxillofacial blunt trauma.

## 4 Conclusion

Avulsion of the globe due to maxillofacial blunt trauma is a rare phenomenon. The primary focus should always be on preserving the globe, whenever possible. Although the globe has several anatomical protective mechanisms, sometimes they fail, and severe injuries, such as traumatic enucleation can occur. There is no standard treatment for this type of injury since they are extremely rare, and prevention is still the best measure to avoid such complications.

## Declaration of Competing Interest

The authors report no declarations of interest.

## Funding

This research did not receive any specific grant from funding agencies in the public, commercial, or not-for-profit sectors.

## Ethical approval

This case report is part of an umbrella research protocol approved by the ethics committee of the University Center of Pará (approval number 524680).

## Consent

Written informed consent was obtained from the patient for publication of this case report and accompanying images. A copy of the written consent is available for review by the Editor-in-Chief of this journal on request.

## Author’s contribution

**Antonia Moraes:** Writing and Data acquisition. **Martha Quaresma:** Writing and Data acquisition. **Thais Silva:** Writing and Data acquisition. **Naama Sousa:** Writing and Data acquisition. **Silvio Menezes:** Conceptualization and Reviewing. **André Ribeiro:** Conceptualization, Writing - Reviewing and Editing. **João Pinheiro:** Writing - Reviewing and Editing.

All authors agree with the publication of the article.

## Registration of research studies

Not applicable.

## Guarantor

Dr André Ribeiro and Dr João Pinheiro are the guarantors of this study.

## Provenance and peer review

Not commissioned, externally peer-reviewed.

## Figures statement

We declare that all identifiable images are essential for this manuscript and informed consent was obtained from the patient for publication.
